# Corrigendum to: Identifying unmet antithrombotic therapeutic need, and implications for stroke and systemic embolism in atrial fibrillation patients: a population-scale longitudinal study

**DOI:** 10.1093/ehjopen/oead023

**Published:** 2023-03-16

This is a corrigendum to: Fatemeh Torabi, Daniel E Harris, Owen Bodger, Ashley Akbari, Ronan A Lyons, Michael Gravenor, Julian P Halcox, Identifying unmet antithrombotic therapeutic need, and implications for stroke and systemic embolism in atrial fibrillation patients: a population-scale longitudinal study, *European Heart Journal Open*, Volume 2, Issue 6, November 2022, oeac066, https://doi.org/10.1093/ehjopen/oeac066

In the originally published version of this manuscript, the second exclusion box in Figure 2 incorrectly read as follows:

31,696 Excluded: Individuals with a death record prior to baseline of 2012-01-01 under 18 at the baseline

This has now been corrected as follows:

4,145,780 Excluded: Individuals who did not meet inclusion criteria of the study

